# Influence of Tobacco Smoking on Public Health

**Published:** 1861-02

**Authors:** 


*Influence of Tobacco Smoking on Public Health.*—Sir CHARLES HASTINGS read a paper on this subject before the National Association for the Promotion of Social Science. He commenced by observing that it might be considered one of the functions of the Association to point out the evil effect on public health of certain active agents in daily use by the community. The one to which he would especially draw attention was tobacco. Tobacco is at present extensively consumed by all classes; it is a very active narcotic agent; its empyreumatic oil acts most deleteriously on the nervous system; and when it is concentrated, death results from it. The author then quoted the account of the chemical nature of tobacco, as given by Professor Johnstone, of Durham, "that the chemical constituents of tobacco are three in number: a volatile alkali, and an empyreumatic oil. The voiatile oil has the odour of tobacco, and possesses a bitter taste. On the mouth and throat it produces a sensation similar to that caused by tobacco smoke. When applied to the nose it occasions sneezing, and when take internally it gives rise to giddiness, nausea, and an inclination to vomit. The volatile alkali has the odour of tobacco, an acrid, burning, long-continuing, tobacco taste, and possesses narcotic and very poisonous qualities. In this latter respect it is scarcely inferior to prussic acid, a single drop being sufficient to kill a dog. Its vapour is so irritating, that it is difficult to breathe in a room in which a single drop has been evaporated. A hundred pounds of the dry tobacco-leaf yield about seven pounds of nicotin. In smoking a hundred grains of tobacco, therefore, say a quarter of an ounce there may be drawn into the mouth two grains or more of one of the most subtle of all known poisons. The empyreumatic oil is acrid and disagreeable to the taste, narcotic and poisonous. One drop applied to the tongue of a cat brought on convulsions, and in two minutes occasioned death. The Hottentots are said to kill snakes by putting a drop of it on their tongues. Under its influence, the reptiles die as instantaneously as if killed by an electric shock. It appears to act nearly in the same way as prussic acid. Experience proves that a large proportion of those who smoke or chew tobacco, do so under the conviction that it is always innocous in its effects, and often beneficial. Now, this is a mistake which the every-day observation of medical practitioners can attest. For among the patients who consult us for various nervous and stomach complaints, it will be found that tobacco smokers form a large proportion. Indeed, we find, unexpectedly sometimes, on inquiry, that the habit of smoking is the source of very distressing aliments, which immediately or gradually subside on omitting the use of this drug. It is grievous to observe that this habit is prevailing among young people, upon whom its effects are most likely to be prejudicial. Strikingly illustrative of this position is the fact, which has been very recently made public, that in the competitive examinations to which young persons are submitted in the military schools in France, the smokers of tobacco occupy the lowest place."

One of the most severe cases of epilepsy which Sir Charles had ever seen was in a boy of twelve years of age, who had been for two years a tobacco-smoker; he recovered only on being prevented from continuing the habit. It could, no doubt be said, and it was true, that thousands pursue this practice without producing epilepsy; but many of these suffer from nervous and digestive disorders.

It was, then, important that the Association should disseminate sound views on the action of tobacco, and should show that this drug cannot be used indiscriminately. An admonition from such a body would come with more force than from the medical profession, whose admnoitions could only find way among the sick and those needing medical care, while the opinion of the Association would permeate the community at large. Sir Charles Hastings theu quoted from the opinion of Sir B. Brodie on the effects of tobacco on the nervous system.

The various institutions now formed and supported for the purpose of diffusing useful knowledge among the laboring classes, ought to be available to assist in this work, if their managers could be made awake to the importance of the question; but, in many instances, these societies are not aware of the baneful action of tobacco on the frame. If they were, smoking-rooms would not form a part of the establishment, whereby the onward progress of civilization is proposed to be insured. It is a sad reflection that it should be considered necessary to insure the attendance of members at a society whose professed object is to advocate civilization by diffusing art and science, that there should be the means supplied for indulging in the evil habit of smoking, as in the clubs of the aristocracy. This Association may at any rate raise a warning voice against such erroneous proceedings, which must doubtless tend to enervate our population, and eventually to produce a degenerate race.

Sir Charles Hastings ended his paper with the following conclusions:

The tthe effects of tobacco-smoking are chiefly dependent upon an empyreumatic volatile oil, and other active principles, whose direct tendency is to act injuriously on the nervous system and digestive organs. That tobacco is extensively consumed by the community, and its use ought to be discouraged. That this Association emphatically records its conviction that societies formed for the purpose of promoting useful knowledge amongst the working classes, should on no account provide smoking rooms for the members.—*British Med. Jour.* Oct. 6, 1860.

